# Gender Differences in Phytoestrogens and the Relationship with Speed of Processing in Older Adults: A Cross-Sectional Analysis of NHANES, 1999–2002

**DOI:** 10.3390/nu11081780

**Published:** 2019-08-01

**Authors:** Jessie Alwerdt, Andrew D. Patterson, Martin J. Sliwinski

**Affiliations:** 1 Center for Healthy Aging, Pennsylvania State University, University Park, PA 16802, USA; 2 Huck Institutes of the Life Sciences, Pennsylvania State University, University Park, PA 16802, USA; 3 The Department of Veterinary and Biomedical Sciences, Pennsylvania State University, University Park, PA 16802, USA

**Keywords:** phytoestrogens, gender differences, cognition, genistein, speed of processing, older adults, aging

## Abstract

Sex hormone changes in adults are known to play a part in aging, including cognitive aging. Dietary intake of phytoestrogens can mimic estrogenic effects on brain function. Since sex hormones differ between genders, it is important to examine gender differences in the phytoestrogen–cognition association. Therefore, the goal of this study is to examine the relationship between urinary phytoestrogens and speed of processing (SOP) and the variation of the association between genders in older adults. Participants were drawn from the 1999–2002 National Health and Nutrition Examination Survey and included 354 individuals aged 65–85 years old. General linear models (GLMs) were used to test for significant gender differences in the relationship between phytoestrogens and SOP. Results from the GLMs showed significant gender differences in the relationship between genistein and SOP. Higher levels of genistein were associated with better SOP in women. This relationship was reversed in men: higher genistein levels were associated with worse performance. Results indicate that there are distinct gender differences in the relationship between genistein and SOP. These results emphasize the importance of considering gender differences when devising dietary and pharmacologic interventions that target phytoestrogens to improve brain health.

## 1. Introduction

Phytoestrogens are involved in many biological activities, such as anti-proliferation and anti-inflammation [1,2], which makes these metabolites a potential candidate target for interventions to mitigate negative cognitive aging effects [3]. For example, several studies have established that neuroinflammation is a major contributor to cognitive decline due to alterations of normal brain functions, such as synaptic plasticity changes and neuronal dysfunction [4,5]. Phytoestrogens have been found to mitigate neuroinflammation by decreasing inflammatory cytokines, such as IL-6, and other associated factors, such a nitric oxide [6,7,8]. Given the robust relationship between estrogen and brain function, metabolites with estrogenic activity from dietary sources deserve further attention [9]. Prior studies have found that phytoestrogens bind to estrogen receptors (ERs) similarly to estradiol, as well as potentially compete with endogenous estradiol via selective estrogen receptor modulator activity (SERM) depending on gender and specific sex hormones [10,11]. With evidence that isoflavones have high affinity to estrogen receptor beta (ERβ) in a manner similar to estradiol, there is potential for phytoestrogens to affect brain activity because ERβ is abundant in the brain and is involved with neural systems [12,13].

Certain diets allow for the regular consumption of phytoestrogens, which are readily abundant in the Mediterranean and Okinawan diets, both of which have been linked to improved cognition [14,15,16,17]. Phytoestrogens are also found in many foods in the Western diet but are not as abundant because of the vast differences in dietary patterns and food choices [18]. Many types of phytoestrogen groups exist, with isoflavones and lignans being the most prevalent. Recommendations for phytoestrogen intake or limitations are little to none in dietary guidelines. Further, the approximate intake by those residing in Western countries is not known. However, given the substantial increase in the production of foods containing soy, it is likely that phytoestrogen intake has substantially increased [19].

Evidence has suggested that the conversion of phytoestrogens is affected by the gut microbiome [20]. For example, it is estimated that approximately 50–60% of Asian populations have the ability to convert daidzein to equol as a result of their gut bacteria, while this percentage is even smaller in Western countries [21,22]. There is evidence that the gut microbiome related to one’s ability to produce equol remains relatively stable, although studies longer in duration are needed [23,24]. To strengthen the understanding of the role of the phytoestrogen–gut microbiome relationship, a recent study demonstrated that phytoestrogens were able to modify the gut microbiome, indicating a bidirectional relationship [25]. Therefore, phytoestrogens may have significant involvement in the gut–brain axis, although the underlying mechanisms are still largely unknown. Given that phytoestrogens can mimic estrogen, have a bidirectional relationship with the gut microbiome, and are able to cross the blood–brain barrier, it is possible that these metabolites have a direct and indirect impact on cognitive function.

A better understanding of the cognition–phytoestrogen association could suggest avenues for addressing age-related cognitive decline. In this paper, we address two fundamental questions: First, we examine whether the type of phytoestrogens affects their relation to cognition, and second, we examine gender differences in this association.

There are lifelong gender differences in sex hormones, which are further complicated by andropause and menopause in the aging population [26,27]. These gender-specific differences in sex hormones may result in gender differences in the phytoestrogen–cognition association, particularly in older individuals. For example, prior studies have demonstrated gender-based variation in neurodegenerative diseases, as well as in normative cognitive aging [28,29,30,31]. More specifically, studies have found gender differences in speed of processing (SOP), a hallmark of cognitive aging [28,31]. Examining the phytoestrogen–cognition association and gender differences, Ostatníková and colleagues [32] demonstrated improved spatial abilities in young men and women after a week of soybean consumption. However, there were evident gender differences in the effect of soybean consumption on basal testosterone (T), salivary T, and plasma estradiol (E2), with an increase in salivary T and plasma E2 only in women. Therefore, phytoestrogens may be sex hormone-dependent and explain the existence of such differences in phytoestrogen intake and its relationship with gender. Further, Gleason and colleagues [33] indicated a significant improvement in cognition in older adult men and women using isoflavone supplementation, although gender differences were not examined. It is important to note that the majority of studies on the phytoestrogen–cognition association have limited their sample population to women only or did not examine gender differences when men were included. Despite robust evidence of gender differences in both sex hormones and cognitive aging outcomes, relatively little research has examined gender differences in the relationship between sex hormones and cognitive function.

The purpose of this study is to examine urinary phytoestrogens and whether gender differences exist between phytoestrogens and SOP, as measured by the Digit Symbol Substitution Test (DSST), in a sample of older adults. This study is a secondary data analysis using data from the National Health and Nutrition Examination Survey (NHANES). Given the complexity of phytoestrogens, analyses examined the overall composition of phytoestrogens, as well as individual phytoestrogens. Analyses also focus on evaluating whether the relationship between the composition and individual phytoestrogen variables differ between men and women.

## 2. Materials and Methods

### 2.1. Participants

Participants in the current study were obtained from the NHANES conducted by the U.S. National Center for Health Statistics for Disease Control and Prevention, specifically for the years 1999–2000 and 2001–2002. For details on the sampling design and the initial protocol, please refer to the published analytical guidelines by Johnson and colleagues [34]. All NHANES protocols and consent to participate were approved and obtained through the NCHS Research Ethics Review Board (ERB). Inclusion criteria for the current study included an age of over 65 with measures of phytoestrogens, cotinine, creatinine, gender, ethnicity, education, poverty–income ratio (PIR), body mass index (BMI), fasting status, and SOP score. The PIR is calculated as family income relative to the poverty threshold. A PIR below 1 is classified as below the poverty line. Fasting status was included to examine the possible influence of samples obtained from non-fasting participants since prior studies have reported that fasting samples are optimal [35]. Participants who had used antibiotics in the past 30 days prior to cognitive testing were excluded from the analysis because prior research has indicated that antibiotic use disrupts the gut microbiome and may interfere with equol production [36]. Previous reports have suggested that smoking interferes with phytoestrogen concentrations. Therefore, cotinine was included as an indicator of smoking status [37]. Due to the potential variation in urine dilution due to the use of spot samples and since prior research has indicated demographic differences in excretion, creatinine was included to correct for these variations [38].

**Figure 1:**
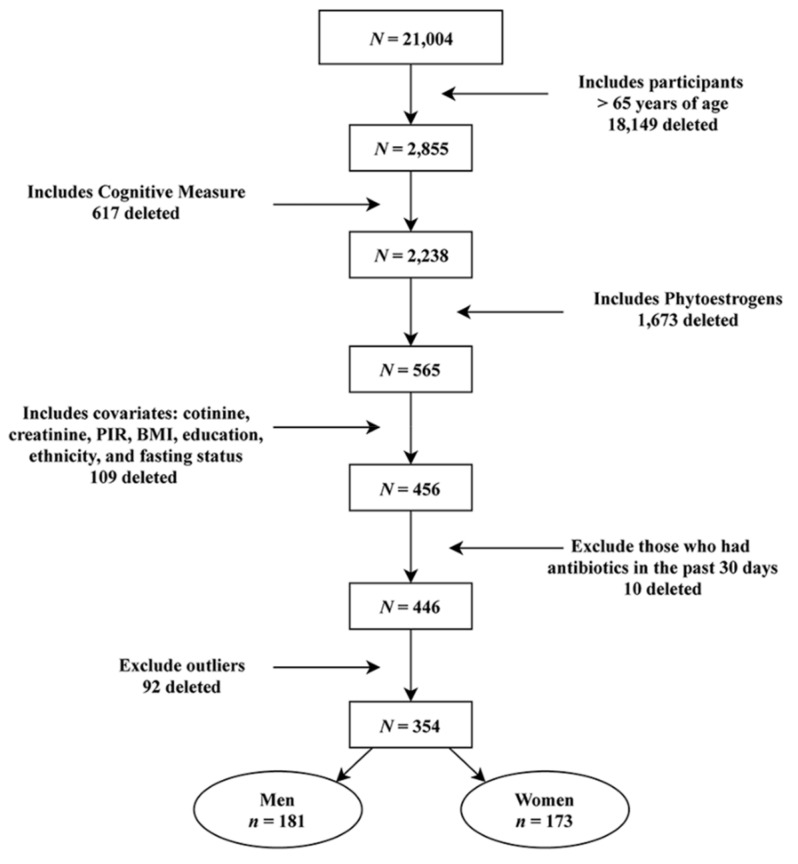
Flowchart based on the inclusion and exclusion criteria to obtain the final sample.

After initial screening, 456 participants met the inclusion criteria: Those who were 65 and older with data on cognition, phytoestrogens, cotinine, creatinine, PIR, education, ethnicity, fasting status, and BMI were included. Then, those who had taken antibiotics in the past 30 days were further excluded (*N* = 10). Lastly, 92 participants were eliminated as outliers. Outliers were determined by examining extreme values of each predictor variable using box and whisker plots, and any outliers that were beyond the 1.5 interquartile range (IQR) on either side of Quartile 1 and Quartile 3 were removed from further analysis. The IQR is a measurement of spread (variability) calculated for each variable separately based on the calculated quartiles [39,40]. Outliers were removed based on the likelihood that these values are measurement error and to improve the distribution normality of the statistical model. The final sample included a total of 354 participants, with 181 men and 173 women (see Figure 1). All data associated with NHANES are publicly available and located in the Center for Disease Control and Prevention repository (URL: https://www.cdc.gov/nchs/nhanes/index.htm).

### 2.2. Measures

Speed of processing (SOP): NHANES data include one cognitive measure that measures SOP using the Wechsler Adult Intelligence Scale, Third Edition (WAIS-III) Digit Symbol Substitution Test (DSST) [41]. This pencil and paper test was timed for 120 s, during which participants were asked to draw the correct symbol that corresponded with the number from the reference key at the top of the page. Five boxes were asked to be completed before the actual timed test was started to ensure that the participant understood the task. Participants were not allowed to skip boxes and were directed to fill in the symbols from left to right. The maximum score that could be obtained was 133. This test is known to have high test–retest reliability and serve as a good marker for age-related studies [42,43,44].

Phytoestrogens: In-spot urine sample data to measure phytoestrogens (O-desmethylangolensin, daidzein, equol, genistein, enterolactone, and enterodiol) were only in a small subset of the NHANES sample. Urine samples were obtained, stored at −20 °C, and analyzed using mass spectrometric (MS/MS) detection at the Division of Environmental Health Laboratory Sciences, National Center for Environmental Health, Centers for Disease Control and Prevention (CDC) under specific guidelines. Quality control measures, limits of detection, and normal ranges for phytoestrogens for this study are explained elsewhere [45,46,47]. Prior studies have indicated that urinary phytoestrogens are reliable biomarkers for representing daily intake and habitual intake of isoflavones and lignans [48,49,50,51].

### 2.3. Statistical Analysis

First, creatinine and all urinary phytoestrogens were log-transformed before any further adjustments. Since there is dilution-dependent sample variation in urine, several adjustments were calculated to obtain error-corrected phytoestrogen values. (1) In the first step, creatinine was adjusted by accounting for covariates that may be related. This was completed by including creatinine as the outcome in a regression model, while all potentially related covariates were included as predictors. Covariates for the creatinine covariate adjustment included age, BMI, ethnicity, smoking, and gender, as suggested by prior research [52,53]. The predicted values (plogC) from the regression model were saved to use in the next step. Prior research has demonstrated that covariate-adjusted creatinine is a more reliable measure for urinary biological measurements than creatinine alone [54,55]. (2) Since the predicted values from the regression model were determined from log-transformed creatinine, the predicted values were exponentiated to prepare for the next step. (3) The final step for preparing creatinine-adjusted phytoestrogen correction included creating a creatinine ratio (observed/plogC = Cratio). Finally, the creatinine ratio could be used for correcting phytoestrogen values. (4) To get the corrected values for phytoestrogens, each phytoestrogen was divided by the creatinine ratio (e.g., daidzein/Cratio). The final calculation resulted in six new phytoestrogen variables that were corrected for dilution-dependent sample variation. These new phytoestrogen variables were used in the main analysis. Demographics and descriptive statistics for the final sample, including age, ethnicity, education, BMI, PIR, creatinine, smoking, fasting status, SOP, raw phytoestrogen values, and adjusted phytoestrogen values are provided in Table 1.

All continuous variables were standardized (mean-centered and z-scored). For the analysis, ethnicity and race were dichotomized to being either non-Hispanic White or other. Education was categorized as either having some college or higher education or having a high school diploma/General Educational Development (GED) certification or less. Cotinine levels, a metabolite to indicate smoking status, were broken into non-smoking (cotinine < 0.05) and smoking (cotinine ≥ 0.05). PIR was dichotomized to indicate whether a subject was below or above the poverty line, according to the Department of Health and Human Services’ poverty guidelines [56]. Fasting status was dichotomized according to whether a participant fasted for 8 h or more.

Using the SAS 9.4 statistical software, a general linear model (GLM) was run that included the covariates, i.e., age, BMI, ethnicity, education, smoking, fasting status, PIR, and creatinine [57,58]. In addition, all individual phytoestrogens were included as predictors, and all phytoestrogen × gender interactions were analyzed to determine whether there were gender differences in the dependent variable, SOP. To plot the interaction, confidence intervals, and scatterplot of the actual data points, the R statistical package “Interactions” was used within the R software [59,60]

## 3. Results

The overall sample size included 354 participants. There were 173 women and 181 men. The breakdown of this sample and exclusion can be found in Figure 1. The participants were approximately 74 years old and mostly non-Hispanic white (~71%). *T*-tests and Chi-square tests were used to determine whether there were any significant group differences between men and women. There were significant gender differences in SOP (t(352) = −2.36, *p* = 0.019), with women achieving higher scores (M = 43.51, SD = 18.48) compared with men (M = 39.11, SD = 16.49). Creatinine levels were significantly different (t(352) = 5.51, *p* < 0.001), with men having higher levels (M = 121.40, SD = 62.02) than women (M = 86.33, SD = 57.56). Group differences were also found in smoking status (X2 (1, *N* = 354) = 7.45, *p* < 0.01), with 46.15% of men as smokers versus 31.98% of women. There were no group differences in any of the phytoestrogens or other covariates. The means, standard deviations, and all significant and non-significant t-test results are reported in Table 1.

**Figure 2:**
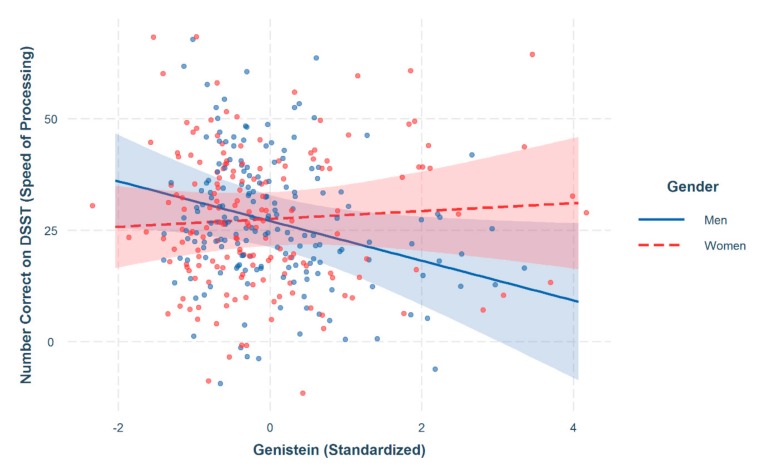
Significant interaction between genistein intake and Digit Symbol Substitution Test Performance (Speed of Processing) for both men and women; 95% confidence intervals. Note: Covariates controlled for included age, BMI, ethnicity, education, smoking, fasting status, poverty-income-ratio (PIR), and creatinine. The scatterplot refers to the actual observed values with each color corresponding to gender. Red dots refer to women and blue dots refer to men.

The GLM with individual phytoestrogens as predictors showed that individual phytoestrogens accounted for 39.82% of the variance in SOP (F [21, 332] = 10.55, *p* < 0.001), and after controlling for the covariates, there was a significant interaction between genistein (as an individual phytoestrogen) and gender (β^1^ = 5.04, t(332) = 2.01, *p* = 0.045). This significant interaction indicated that higher levels of genistein in women were associated with better SOP performance, whereas in men, higher levels were associated with worse SOP performance (see Table 2 and Figure 2). No other significant effects of the individual phytoestrogens or their interactions with gender were observed.

## 4. Discussion

Results from the current study indicate that higher levels of genistein were associated with better SOP performance in women but with lower performance in men. None of the other phytoestrogens were associated with SOP performance or interacted with gender. Therefore, the results pertaining to genistein are discussed further.

Very few studies have specifically looked at gender differences in relation to genistein and cognitive function, so comparing the present results with the literature is difficult. There has, however, been more extensive research on soy supplementation, which is relevant to this study since soy products contain a high abundance of genistein. Further, the majority of these studies have included only postmenopausal women. Within these studies, the overall results align with our finding that genistein is associated with improved cognitive performance in older women [33,61,62,63,64,65]. Two studies, both of which were randomized controlled trials that administered isoflavones through supplementation, reported results that are contrary to our findings since they indicated no cognitive improvement in any cognitive domains in postmenopausal women [66,67]. Although this study was observational, these results continue to support the positive association that suggests soy consumption benefits cognitive performance in older women.

Studies involving men have been limited compared with studies involving women. In addition, these studies lack an analysis of older subjects. Consistent with our findings that genistein was associated with worse cognitive performance in men, White and colleagues [68] found that cognitive performance declined from mid-life to late-life in Japanese men who consumed more tofu. However, this study differed in the way by which phytoestrogens were measured; data were collected using a food frequency questionnaire rather than biological measurements. Further, brain atrophy in men determined by brain weight at autopsy and brain imaging was seen in men who consumed more tofu from mid-life to late-life. Further, Lephart and colleagues [69] and Lund and colleagues [70] both indicated worse cognitive performance in visual–spatial memory in soy-fed male rats, while female rats improved in cognitive performance. Additionally, the sexually dimorphic brain regions in male rats were increased significantly compared with male rats that did not consume soy, while the opposite relationship was found in female rats [69].

Although the longitudinal study conducted by White and colleagues [68] is one of the strongest to date that involves men and supports our results, there have been studies indicating that soy consumption improves cognitive function in men. Recently, a large cross-sectional study involving both older adult Taiwanese men and women indicated that higher consumption of soy foods resulted in better cognitive performance when grouped, but there were no gender differences [71]. Although the study conducted by Lin and colleagues is similar to our study given its cross-sectional design, our study used urinary phytoestrogens measured by LC/MS, while Lin and colleagues used dietary recall measures to estimate the actual soy intake of the sample. The studies that have been discussed thus far have either involved soy supplementation or measurement estimation from dietary intake, which strengthens the contribution of our study. Further, Lin and colleagues [71] utilized logistic regression to compare those who showed cognitive impairment with those who did not. In a double-blind placebo-controlled trial including only men, Thorp and colleagues [72] found results contrary to ours. Specifically, they found an improvement in visual–spatial memory in those who were given 12 weeks of soy supplementation. Although a double-blind placebo-controlled trial is the gold standard, there was a vast age difference in the sample (30–80-year age range), and the sample size only consisted of 34 participants. Two other studies with results that differ from our findings included young adults who were given soy supplementation that improved executive function, as well as spatial cognitive performance [73,74]. Both studies had a very small sample size, especially the study by Celec and colleagues [73] with a sample of only 7 participants. Additionally, no biological measurements were taken to determine differences in the types of phytoestrogens or how the supplement is absorbed.

All the prior studies differ in many ways compared with the current study; for example, there is variation in the way that phytoestrogen intake was assessed. It is important to stress the difference between measuring phytoestrogens by self-reported dietary intake and utilizing biological parameters measured by LC/MS, which gives absolute quantities versus estimations. To our knowledge, this is the first study to look at gender differences in urinary phytoestrogens rather than relying on self-reported dietary intake. A strength of using urinary phytoestrogens is the ability to examine individual phytoestrogens and their association with cognitive performance. Gender differences specific to genistein would have otherwise gone undetected. Further, genistein has been found to increase neurobiological activity via ERβ more than other phytoestrogens [75,76]. Additionally, urinary phytoestrogens have been shown to remain stable in individuals by collecting two separate urine samples on separate days [77]. Several studies have demonstrated that urinary phytoestrogens have also predicted one’s habitual intake of phytoestrogens and remain relatively stable in adolescents. These findings are from a longitudinal study involving urinary phytoestrogens and dietary intake over many years [50,78,79,80,81]. On the basis of these results, further studies should be pursued in an attempt to untangle phytoestrogen differences, given that each phytoestrogen may influence cognition differently.

The present study has several limitations that should be highlighted. First, the NHANES data set contained only a single measure of cognition, the DSST. Although the measure of cognition is referred to as SOP in this study, prior studies have suggested that the DSST measure taps multiple domains, including SOP, psychomotor speed, and memory, which complicates the isolation of the specific aspect of cognition that might be affected by genistein [82,83,84]. Second, because of the cross-sectional nature of this data, change and causation cannot be determined. Future research should include longitudinal data that measure variations in sex hormones and the interaction and change involving phytoestrogens. Although prior studies have demonstrated that urinary phytoestrogens are considered reliable as biomarkers of these metabolites, there is still a lack of longitudinal studies involving multiple measurements of cognitive performance and phytoestrogen intake, especially in older adults. Since phytoestrogens mimic estrogenic activity, it is important to identify changes in sex hormones in relation to phytoestrogen intake. Third, it is evident that the gut microbiome may significantly affect the conversion and bioavailability of the phytoestrogens consumed [85]. Recently, genistein was found to be associated with improvement in both the gut microbiome and cognitive decline and counteracted the deleterious impact of a high-fat diet in mice [86]. Therefore, accounting for the gut microbiome in the phytoestrogen–cognitive relationship may demonstrate a source of variation.

## 5. Conclusions

In conclusion, differences are evident in the relationship between genistein and SOP depending on gender in older adults while controlling for age, BMI, ethnicity, education, smoking, fasting status, PIR, and creatinine. These results warrant further research to determine gender differences in the effects of phytoestrogens on cognitive function and brain health. Clarification of the precise mechanism that explains these gender differences is strongly needed.

## Figures and Tables

**Table 1:** Demographic Characteristics.

	All		Men		Women	
	*N* = 354		*N* = 181		*N* = 173	
**Characteristic**	**M (%)**	**SD**	**M (%)**	**SD**	**M (%)**	**SD**
Age (years)	74.10	6.26	73.5	6.03	74.74	5.46
Non-Hispanic White	(71.19)		(70.88)		(71.51)	
Education	(40.06)		(39.56)		(40.12)	
Body Mass Index (BMI)	27.41	4.57	27.39	4.13	27.43	5.01
Above Poverty Line (PIR)	(84.46)		(84.62)		(84.30)	
Creatinine (mg/dL) *	104.38	62.34	121.40	62.02	86.33	57.56
Smoke *	(39.27)		(46.15)		(31.98)	
Fasted	(63.28)		(66.48)		(59.88)	
Speed of Processing *	41.25	17.60	39.11	16.49	43.51	18.48
Raw Scores (ng/mL)						
O-DMA	17.85	67.67	18.54	43.78	17.13	86.16
Daidzein	115.09	249.41	145.10	312.20	83.37	152.60
Equol	10.14	11.68	10.68	11.56	9.57	11.81
Genistein	64.89	130.26	70.22	141.00	59.25	117.90
Enterolactone	646.24	854.33	713.50	993.60	575.10	672.20
Enterodiol	58.15	71.90	59.60	72.54	56.61	71.40
Adjusted						
O-DMA	0.68	2.65	0.75	2.80	0.62	2.49
Daidzein	3.83	2.83	4.10	2.90	3.54	2.73
Equol	2.02	1.43	2.06	1.43	1.98	1.43
Genistein	3.34	2.34	3.47	2.13	3.20	2.54
Enterolactone	6.84	4.61	6.97	6.25	6.72	6.07
Enterodiol	3.93	2.50	3.99	3.62	3.87	3.49

Note: * significant gender differences; Adjusted = urinary phytoestrogen values that were corrected by creatinine to account for dilution-dependent sample variation in urine; O-DMA = O-desmethylangolensin.

**Table 2:** Gender Differences in Phytoestrogen Intake and Speed of Processing Performance in Older Adults.

**Predictor**	**Estimate**	**Std. Error**
Constant	29.01 ***	2.84
Age	−5.19 ***	0.82
BMI	1.25	0.84
Ethnicity	−9.49 ***	1.99
Education	9.61 ***	1.64
Smoking	−2.52	1.61
Poverty–Income Ratio	11.10 ***	2.23
Creatinine	−3.01	2.15
Fasting status	2.17	1.61
Women	2.96	2.13
O-Desmethylangolensin	0.84	1.24
Daidzein	−0.26	2.04
Equol	−0.22	1.52
Genistein	−4.02 *	2.00
Enterolactone	2.28	2.25
Enterodiol	−1.03	1.82
**Predictor (Interactions)**		
O-Desmethylangolensin × Women	−0.58	1.82
Daidzein × Women	−0.79	2.63
Equol × Women	−0.42	2.02
Genistein × Women	5.04 *	2.51
Enterolactone × Women	−0.04	2.88
Enterodiol × Women	−3.06	2.45

Note: All urinary phytoestrogen values were adjusted by creatinine to account for dilution-dependent sample variation in urine; all continuous variables were centered; ethnicity (0 = white, 1 = non-white), education (0 = high school or less, 1 = more than high school), smoking (0 = non-smoking, 1 = smoking), and PIR (0 = below poverty, 1 = above poverty level) were all dummy coded; PIR = poverty–income ratio, BMI = body mass index. * *p* < 0.05, *** *p* < 0.001.

## Abbreviations

BMI	body mass index
CDC	lefts for Disease Control and Prevention;
DSST	Digit Symbol Substitution Test
E2	plasma estradiol
ERβ	estrogen receptor beta
GED	General Educational Development
GLM	general linear model
NHANES	National Health and Nutrition Examination Survey
SOP	speed of processing
PIR	poverty–income ratio
T	testosterone
